# Evaluation of point‐of‐care diagnostics for sexually transmitted infection on oral PrEP initiation and persistence among young people in South Africa: a randomized controlled study

**DOI:** 10.1002/jia2.26488

**Published:** 2025-05-12

**Authors:** Dvora Joseph Davey, Lauren Fynn, Elzette Rousseau, Pippa Macdonald, Bryan Leonard, Keitumetse Lebelo, Ande Kolisa, Francesca Little, Linda‐Gail Bekker

**Affiliations:** ^1^ Division of Infectious Diseases Geffen School of Medicine University of California Los Angeles California USA; ^2^ Desmond Tutu HIV Centre Institute of Infectious Diseases and Molecular Medicine University of Cape Town Cape Town South Africa; ^3^ Department of Epidemiology and Public Health School of Public Health University of Cape Town Cape Town South Africa; ^4^ Department of Statistical Sciences University of Cape Town Cape Town South Africa

**Keywords:** sexually transmitted infections, pre‐exposure prophylaxis, HIV prevention, Africa

## Abstract

**Introduction:**

Pre‐exposure prophylaxis (PrEP) services are linked to increased sexually transmitted infection (STI) diagnoses, which may facilitate PrEP uptake. We hypothesized that point‐of‐care (POC) STI testing and treatment would improve PrEP initiation and persistence.

**Methods:**

Between September 2023 and November 2024, we conducted a single‐centre, open‐label, unblinded, randomized controlled trial among adolescent girls and young women (15−29 years old) or male partners (any age). Participants were randomized 1:1 to standard syndromic STI management (SOC) or POC testing for *C. trachomatis*, *N. gonorrhoeae*, syphilis and *T. vaginalis* (women only). All participants received standard HIV prevention counselling, including the offer of oral PrEP. The primary outcome was effect of POC STI testing versus syndromic management on PrEP initiation; secondary outcomes included persistence at 1 and 4 months (PrEP prescription), verified in the secondary analysis of tenofovir diphosphate (TFV‐DP) in dried blood spots (DBS) in a random subset. TFV‐DP in DBS was analysed in a subset. Analysis was intention‐to‐treat, adjusted for age and sex.

**Results:**

We enrolled and randomized 900 participants (452 in intervention; 448 in SOC). The mean age was 20.4 years (SD = 4.2); 48% were female. In the intervention arm, 435 received POC STI testing (96%); 25% (110 of 435 tested) were diagnosed with =>1 STIs; 84% were treated. In SOC, 7% of participants reported symptoms of STIs (31); 88% were treated (27). Overall, 64% of participants in SOC versus 62% in intervention‐initiated PrEP (RR = 0.98, 95% CI = 0.88ng women and partners1.08). In the intervention, 41% persisted on PrEP at 1 month and 25% through 4 months, compared to 46% and 19%, respectively, in SOC (aRR intervention = 1.39; 95% CI = 0.93−2.09; *p* = 0.08). In participants treated for STIs or syndromically, 77% initiated PrEP versus 60% untreated/diagnosed (aRR = 1.14; 95% CI = 1.02−1.27); 19% versus 14% persisted on PrEP at 4 months (aRR STI/syndrome treated = 1.41; 95% CI = 0.79−2.51). Overall, 30% of 64 DBS had any TFV‐DP levels present with no difference by study arm (RR = 0.74; 95% CI: 0.38−1.41).

**Conclusions:**

POC STI testing did not increase PrEP initiation or 1‐month persistence but showed a moderate association with 4‐month persistence. STI treatment (syndromic or confirmed) was linked to higher PrEP uptake and persistence. Integrating STI management may improve PrEP persistence among youth.

## 1 INTRODUCTION

Sexually transmitted infections (STIs) remain a critical public health issue, particularly in the context of HIV prevention [1, 2, 3, 4]. The intersection between HIV and STIs is particularly concerning, as other STIs are recognized as significant risk factors for HIV acquisition [1, 5, 6, 7]. This relationship is most pronounced in Africa [4, 8]. Improving diagnosis and treatment of STIs is a critical issue in HIV prevention, including how best to integrate STI care into HIV prevention programmes. Syndromic management is the standard of care (SOC) in low‐ and middle‐income countries due to resource limitations, but fails to detect asymptomatic infections and may lead to overuse of antibiotics, contributing to antibiotic resistance [2, 9, 10, 11].

Pre‐exposure prophylaxis (PrEP) uptake has been slow in many high‐burden settings in Africa, and among those who do initiate, there is a remarkably high non‐return rate for the first month's visit to replenish PrEP supplies, with a further decline in the fourth month. Several studies have demonstrated high incidence and prevalence of STIs in those taking oral PrEP due to potential risk compensation, or decreased condom use by PrEP users [12]. While there are increasing reports of high rates of STI diagnosis in individuals accessing PrEP services, individuals who are diagnosed with STIs may be more inclined to initiate and persist on PrEP. Thus, integrating STI and HIV prevention services can act as a critical touchpoint for individuals to become aware of their STI and HIV risks, providing an entry point for initiating and persisting on oral PrEP [6, 13, 14, 15].

Community‐based STI testing and management, integrated within HIV and sexual and reproductive health (SRH) services, shows promise in improving access and reducing STI prevalence [14, 16, 17, 18]. In addition, the development and integration of point‐of‐care (POC) STI testing within SRH services are crucial for effective STI control and improved health outcomes. The World Health Organization's (WHO) 2022–2030 strategy advocates for the integration of STI and HIV responses, emphasizing the need to target populations with high prevalence or incidence of STIs, including adolescents and young people and pregnant women [7, 16]. Innovative, low‐cost POC STI testing offers same‐day accurate results compared with syndromic management of STIs and can potentially transform STI control by improving the diagnoses and treatment among those with both symptomatic and asymptomatic STIs, which are common, especially in women.

Combining STI care into PrEP programmes can create a more holistic approach to SRH, promoting early detection and treatment, and reducing the overall burden of STIs and HIV. Recent studies indicate a high prevalence and incidence of STIs among young adults using PrEP in South Africa, with chlamydia being the most common (25.7% in females, 20.0% in males) [11] and STI incidence rates reaching up to 42 per 100 person‐years [17, 18]. The high proportion of asymptomatic infections (98%) underscores the limitations of syndromic management, emphasizing the need for enhanced STI screening and integration into PrEP programmes [11, 17]. In the FastPrEP‐STI study, we evaluated the effect of STI POC testing and treatment on PrEP uptake and persistence among young people. In this study, we hypothesized that POC STI testing would lead to higher PrEP initiation and persistence compared with SOC STI syndromic testing in the randomized controlled trial. As a post hoc analysis, we also explored whether any STI diagnosis and management (whether based on aetiology or syndromes) might lead to enhanced PrEP uptake and persistence among young people accessing community‐based sexual and reproductive services.

## 2 METHODS

### 2.1 Study population

The FastPrEP‐STI study was a single‐site, open‐label, unblinded, randomized controlled study in Cape Town, South Africa, which enrolled 900 eligible young people at an initial health service visit in one mobile community clinic. Our study was nested as a sub‐study within the expansive ongoing FastPrEP (FP) implementation programme that offers HIV testing, SRH services and oral PrEP (TDF/FTC) counselling and prescriptions as part of an integrated SRH package for adolescent girls and young women and male partners through mobile vans, public health clinics and FP depots in the community [18]. FP delivers comprehensive SRH services, including HIV prevention via PrEP, to all young people. Utilizing a hub‐and‐spoke model [18], the initiative enhances PrEP uptake through accessible, community‐based service points, user‐centred delivery options and biometric data tracking to optimize intervention efficacy and adherence.

In this study, participants were recruited from a single selected FP mobile community van. Participants were eligible to participate if they presented at the clinic for any health services and were: (1) adolescent girls and young women (15−29 years old) or male partners (any age); (2) sexually active in the last 3 months; (3) confirmed HIV negative through a fourth‐generation antigen and antibody rapid HIV test; (4) without psychiatric or medical contraindications to PrEP; (5) able and willing to consent to participate in the study. We hypothesized that POC STI testing and treatment would improve PrEP initiation and persistence.

### 2.2 Study procedures

To evaluate the effect of STI testing and treatment on PrEP initiation and persistence, we enrolled participants at baseline who were randomized 1:1 to either the SOC syndromic STI management or the intervention (POC STI testing and treatment). The study Data Manager generated the random allocation sequence using dynamic permuted blocks within REDCap. The study Research Assistants enrolled the participants and assigned the participants to the interventions per the REDCap allocation. Participants were followed through 4 months and invited to return for 1‐ and 4‐month study visits (Table 1). If the participants initiated PrEP, they received a 1‐month supply at baseline and then a 3‐month supply at the subsequent visit from the study nurse. Study interviewers collected data on the participant demographics, sexual behaviours, prior HIV testing (and partner serostatus), STI symptoms and treatment behaviours (and preferences for treatment and partner notification), as well as interest in initiating PrEP. We collected data on PrEP prescriptions and STI treatments from the study nurse's data collection in the participants’ medical records.

**Table 1:** Description of intervention and standard of care in FastPrEP‐STI randomized control study in Cape Town, South Africa

	Standard care arm	Intervention arm
**Baseline**	Eligibility screening and informed consent Survey Syndromic STI questionnaire and treatment PrEP counselling for those who initiate (standard)	Eligibility screening and informed consent Survey Vaginal swab (self‐collected) or urine sample (males) STI point‐of‐care testing for CT/NG and syphilis, TV tested in women, with treatment PrEP counselling for those who initiate (standard)
**Month 1**	PrEP counselling (standard) and PrEP prescription (if want to start or continue) Dried blood spot collection among those on PrEP Survey	PrEP counselling (standard) and PrEP prescription (if want to start or continue) Dried blood spot collection among those on PrEP Survey
**Month 4**	PrEP counselling (standard) Dried blood spot collection among those on PrEP Survey	PrEP counselling (standard) Dried blood spot collection among those on PrEP Survey

Abbreviations: CT, *Chlamydia trachomatis*; NG, *Neisseria gonorrhoeae*; PrEP, pre‐exposure prophylaxis; STI, sexually transmitted infection; TV, *Trichomonas vaginalis*.

For the SOC arm, participant treatment was based on the participant reporting symptoms and a trained nurse identifying consequent STI syndromes, which were treated according to South African national guidelines [19]. Syndromes included vaginal discharge, urethral discharge, pain in urination or pain during sex. In the intervention arm, participants were asked to self‐collect a urine sample (male) or vaginal swab (female) and samples were then tested for *C. trachomatis* (CT) or *N. gonorrhoeae* (NG) with Cepheid GeneXpert test (results available in 90 minutes). In addition, participants were tested using rapid Duo HIV and syphilis test (manufactured by Abbott, results available in 15 minutes), followed by rapid plasma reagin (RPR) testing for positive rapid test in the lab (results available in 1–3 days). In addition, we tested women's vaginal swab samples for *T. vaginalis* using the OSOM Trichomonas Rapid Test Kit (Sekisui Diagnostics, Cambridge, MA, USA) with results available in 15 minutes. Participants in the SOC arm who reported having an STI syndrome were treated immediately. Participants in the intervention arm were encouraged to wait for their results, and those diagnosed with an STI received same‐day treatment and a partner notification letter for their partner to access treatment. STI treatment was based on the South African national guidelines.

At the 1‐ and 4‐month follow‐up study visits, participants reported on PrEP adherence in the past week and month. PrEP persistence was measured as receipt of a repeat PrEP prescription by the study nurse within 28 days of the study visit. In addition, we collected dried blood spots (DBS) from individuals who reported taking any PrEP in the last 30 days to evaluate objective levels of tenofovir diphosphate (TFV‐DP) in all participants. Analysis of the DBS for TFV‐DP was conducted among a random subset of 20% of samples at 1 and 4 months (from each arm). Qualitative analysis of TFV‐DP was conducted at the University of Cape Town Division of Clinical Pharmacology.

### 2.3 Analysis

Our primary outcome was the effect of the intervention (STI POC testing vs. syndromic management of STIs) on PrEP initiation (receipt of PrEP prescription at baseline). We also evaluated the effect of the intervention on PrEP persistence, defined as receipt of a repeat PrEP prescription at 1 and 4 months following PrEP initiation at baseline.

Primary analyses were by intention‐to‐treat. Those who did not receive the POC STI test were treated as part of the intervention arm of the study. Participants who did not return for the follow‐up study or PrEP collection visit were assigned as discontinued on PrEP (not persistent). We also evaluated secondary study exploratory outcomes using: (1) per‐protocol analysis of receipt of a POC STI test versus syndromic management at baseline (instead of study arm allocation); (2) the effect of STI treatment (in POC or syndromic management arms); and (3) STI treatment in the intervention arm.

We examined baseline demographic and behavioural characteristics by study arm using means standard deviations (SD) for continuous variables, and frequencies and proportions for categorical variables. Wilcoxon rank‐sum (for continuous variables), Chi‐squared and Fischer's exact tests (for categorical variables) were used to explore the primary and secondary outcome variables. One‐sided *p*‐values were considered significant if <0.05.

We used binomial regression models to examine the predictors of outcomes of interests for primary and secondary outcomes. Model results will be presented as crude risk ratios (RR) and age and sex adjusted RRs (as a priori confounders) with 95% confidence intervals (CIs). All statistical analyses were conducted with STATA v.15 (College Station, TX: StataCorp LLC).

### 2.4 Sample size

For the primary objective, we hypothesized that the intervention arm would lead to improved PrEP initiation and persistence at 1 and 4 months compared to SOC syndromic STI management. Based on our previous work among a different population of pregnant women (in clinical care), we estimated that PrEP persistence would be 70% in the intervention arm compared to 55% in the SOC arm. To achieve 80% power of detecting a risk ratio of persisting on PrEP of 1.3, we enrolled 900 participants.

### 2.5 Ethics

The FastPrEP‐STI study was approved by the Human Research Ethics Committee at the University of Cape Town (#233/2023) nested in the larger Fast‐PrEP study (#713/2021). Written informed consent was provided by all participants in their preferred local language. Participants who were 15–17 years old had a waiver of parental consent. Participants were reimbursed R150 (approximately $8 USD) in a cash voucher at each study visit.

## 3 RESULTS

### 3.1 Demographics

**Figure 1:**
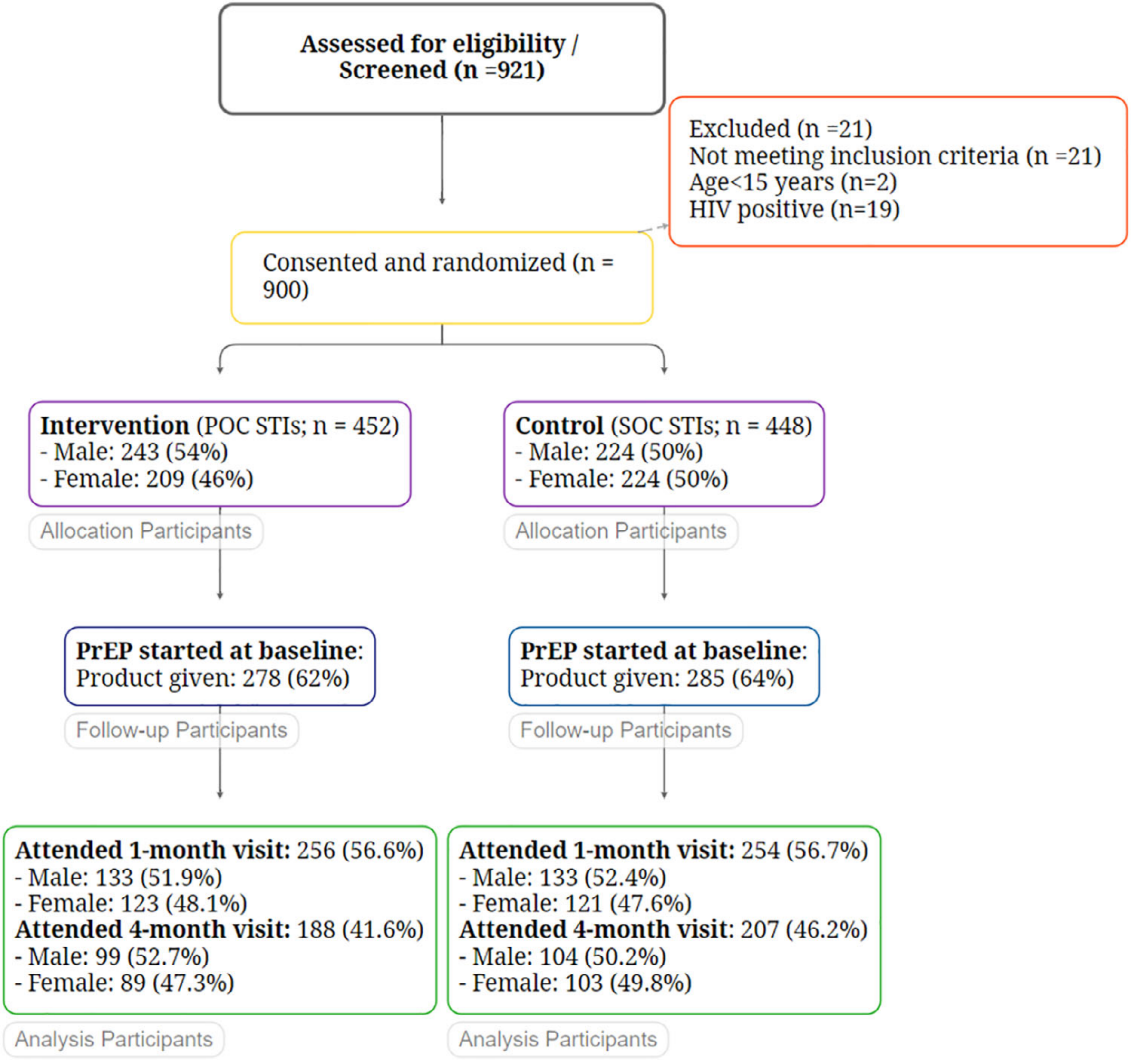
CONSORT Flow diagram for FastPrEP STI sub‐study in Cape Town, South Africa (September 2023−May 2024). Abbreviations: POC, point‐of‐care; PrEP, pre‐exposure prophylaxis; SOC, standard of care.

Between September 2023 and May 2024, we screened 921 people for eligibility. Overall, 21 people did not meet inclusion criteria due to HIV‐positive diagnosis (*n* = 19) or because they were underage (<15 years old; two people) (Figure 1, Consort Diagram). We enrolled 900 participants of which 50.2% were randomized to the intervention (*n* = 452) and 49.8% to the SOC (*n* = 448) arm. There were no differences in socio‐demographic nor behavioural characteristics between the two study arms. We followed participants for 4 months (4 months + 1 month for outcome ascertainment) through November 2024.

The mean age across both arms was 20.4 years (standard deviation = 4.2). Overall, 48% of participants were female (*n* = 433) and participant sex was distributed similarly in both study arms (50% in SOC and 48% in intervention arm). Two women were pregnant (0.5%). Over three‐quarters reported HIV testing in the past 6 months (78%) and 8.1% were married or cohabiting with their partner. Overall, 1.6% reported having a partner who was living with HIV, and 64% reported condomless sex at the last sex. One‐fifth of participants reported having a casual sex partner, and 64% of women reported current hormonal contraceptive use (Table 2).

**Table 2:** Socio‐demographic and health data at baseline in FastPrEP STI study, Cape Town, South Africa

	Intervention (*n* = 452)	Standard of care (*n* = 448)	Total (*n* = 900)
Age (mean; SD)	20.1 (4.2)	20.7 (4.3)	20.4 (4.2)
Sex (female at birth)	209 (46%)	224 (50%)	433 (48%)
Prior HIV testing	346 (77%)	359 (80%)	705 (78%)
Relationship status (married or cohabiting vs. not)^a^	39 (9.7%)	34 (8%)	73 (8.1%)
Partner living with HIV^a^	6 (1.5%)	8 (2%)	14 (1.6%)
Hormonal contraceptive use^b^	75 (68%)	64 (60%)	139 (64%)
Condomless sex at last sex	293 (65%)	284 (63%)	577 (64%)
IPV reported (ever)	40 (11%)	51 (11%)	101 (11%)
Casual sex partner reported (current)	89 (20%)	89 (20%)	178 (20%)
STI symptoms reported (in past month)	93 (21%)	105 (23%)	198 (22%)
STI treatment given	94 (84%)^c^	27 (88%)^d^	115 (13%)
STI diagnosed (intervention only)	106 (24%)	N/A	N/A

*Note*: Variables are group *n* (%) unless otherwise indicated.

Abbreviations: IPV, intimate partner violence; POC, point‐of‐care; SD, standard deviation; SOC, standard of care; STI, sexually transmitted infection.

^a^ Among those who reported having a partner (*n* = 404 in standard care arm and 404 in intervention arm).

^b^ Among women (*n* = 217 responded of 433 women in study).

^c^ Among those who were diagnosed with an STI (*n* = 110 of 435 tested).

^d^ Among those who reported syndromes to study nurse (*n* = 31, 7% of standard of care arm).

### 3.2 STI symptoms and syndromic management

In the SOC, 7% of participants reported symptoms consistent with nurse‐confirmed STI syndromes (*n* = 31) including vaginal or urethral discharge, pain in urination, ulcers or pain in sex. Overall, 27 of 31 (88%) were treated for a current symptom of an STI by the study nurse; four participants were referred by the nurse for clinical care including herpes treatment (unavailable on the mobile unit).

### 3.3 STI diagnosis and treatment

In the intervention arm, 96% (*n* = 435 of 452) received POC STI testing and 25% (110 of 435 tested) were diagnosed with one or more STIs (34% females; 18% males, *p*<0.001). Overall, 92 participants diagnosed with one or more STIs were treated for the STI (84%), others did not wait for their STI results and did not return for treatment. STI diagnoses included: 17% were CT+ (73; 92% treated); 8% were NG+ (35; 94% treated); 4% were TV+ (10; 100% treated); 6.1% were rapid syphilis+ (27; 10% in females and 3% in males, *p* = 0.01). Following a positive rapid test, samples were tested for RPR titres and 21 were RPR+ in subsequent laboratory analysis (78%, 22 were treated, 82%). Overall, 21% of participants (93 of 452) in the intervention arm reported recent STI symptoms of which 30% (32) tested gene Xpert positive for an STI.

### 3.4 PrEP initiation

Overall, 62% (95% CI = 58.8, 65.1) of participants initiated PrEP at baseline (*n* = 558), with no significant difference between study arms (64% in SOC [95% CI = 56.9, 65.9] vs. 62% in the intervention [95% CI = 59.1, 67.9]; RR = 0.98, 95% CI = 0.88, 1.08; age and sex adjusted [aRR] = 0.97, 95% CI = 1.05, 1.28) (Table 3).

**Table 3:** Evaluation of PrEP initiation and continuation (return and receipt of repeat prescription) at 1 month and through 4 months follow‐up in FastPrEP STI study

Outcome (by study arm)	Standard care (*n* = 448)	Intervention (*n* = 450)	Risk ratio (95% CI)	Adjusted risk ratio (95% CI)^a^
**Intention to treat analysis (by study arm allocation)**				
**Oral PrEP initiation at baseline**				
Initiated PrEP	285 (64%)	278 (62%)	0.98 (0.88, 1.08)	0.97 (0.95, 1.28)

**PrEP continuation (of *n* = 563 on PrEP)**:	**Of *n* = 285 who started PrEP**	**Of *n* = 278 who started PrEP**		
Persistence on PrEP at 1 month	130 (of 285; 46%)	114 (of 278; 41%)	0.83 (0.59, 1.16)	0.83 (0.59, 1.16)
Persistence on PrEP through 4 months^b^	55 (of 285; 19%)	69 (of 278; 25%)	1.38 (0.93, 2.06)	1.39 (0.93, 2.09)

*Note*: No return for study visit is equivalent to discontinued PrEP for this study definition.

Abbreviations: CI, confidence interval; PrEP, pre‐exposure prophylaxis.

^a^ Adjusted for participant age and sex.

^b^ Persisted at both 1‐ and 4‐month visits; confirmed with repeat prescription, through to 4 months.

### 3.5 PrEP persistence

Overall, 43% (95% CI = 39.3, 47.5) of those who initiated PrEP at baseline (*n* = 563) persisted at 1 month with no difference by study arm (46% in SOC [95% CI = 39.9, 51.4] and 41% in intervention [95% CI = 35.4, 46.9], RR = 0.83, 95% CI = 0.59, 1.16). Through 4 months, 25% of people in the intervention arm (95% CI = 20.1, 30.2) persisted on PrEP compared with 19% in the SOC arm (95% CI = 20.1, 30.2; RR = 1.38, 95% CI = 0.93, 2.06, aRR = 1.39, 95% CI = 0.93, 2.09). See Table 3 for persistence in the overall study by study arm.

We analysed a random selection of 20% of DBS for analysis of TFV‐DP among participants who returned for their study visits and reported to have used PrEP in the last 30 days. Among the 377 DBS samples collected, we submitted 75 to the lab for analysis. Overall, the lab was able to analyse 64 DBS samples (85%). Overall, 30% [19] had TFV‐DP present above the threshold of 16.6 fmol/3 mm punch. In the control group, 12 out of 37 DBS samples (32%) had TFV‐DP present, compared to 7 out of 32 DBS samples (22%) in the intervention group (RR = 0.74, 95% CI: 0.38−1.41, *p* = 0.35), suggesting that there was no difference in PrEP adherence per TFV‐DP levels by study arm.ad

### 3.6 Post‐hoc analyses

In the per‐protocol analysis of those who received STI POC versus syndromic management in the study, there were no differences in PrEP initiation (63% vs. 65% initiated, respectively, aRR = 0.85, 95% CI = 0.65, 1.15). Among participants who were treated for an STI (based on POC diagnosis or syndromic management), 77% (96 of 125) initiated PrEP compared to 60% (467 of 773) in not treated/diagnosed group (RR = 1.25, 95% CI = 1.12, 1.40), which remained significant after adjusting for age and sex (aRR = 1.14, 95% CI = 1.02, 1.27). Among those treated in the POC arm, 76% initiated PrEP compared (83 or 110) with 57% in those untreated (193 of 540; aOR = 2.17, 95% CI = 1.30, 3.62) (Table 4). Among participants who were treated for an STI after POC diagnosis, or syndromic management, compared to those who were not diagnosed or not treated for an STI were moderately more likely to persist on PrEP through 4 months (RR = 1.43, 95% CI = 0.80, 2.54), but not 1 month (RR = 1.02, 95% CI = 0.66, 1.59). No other STI treatment nor diagnosis variables were associated with PrEP persistence (Table 4).

**Table 4:** Secondary analyses of PrEP initiation and continuation at 1 month and 4 months by STI diagnosis and treatment in FastPrEP

	Untreated or undiagnosed with STI (*n* = 775)	Treated for STIs or syndromic treatment (*n* = 125)	Risk ratio (95% CI)	Adjusted risk ratio (95% CI)^a^
**1. Evaluation of STI diagnosis treatment or syndromic treatment (both study arms, *n* = 900)**				
Initiated PrEP at baseline	467 (60%)	96 (77%)	**1.25 (1.12, 1.40)**	**1.14 (1.02, 1.27)**
Persisted on PrEP 1 month	202 (of 467; 43%)	42 (of 96; 44%)	1.02 (0.66, 1.59)	0.98 (0.63, 1.53)
Persisted on PrEP through 4 months	65 (of 467; 14%)	18 (of 96; 19%)	1.43 (0.80, 2.54)	1.41 (0.79, 2.51)

	**Undiagnosed or untreated for STI (*n* = 340)**	**Diagnosed and treated for STI (*n* = 110)**		
**2. Evaluation of STI diagnosis and treatment in intervention arm (*n* = 450)**				
Initiated PrEP at baseline	193 (57%)	83 (76%)	**2.31 (1.43, 3.76)**	**2.17 (1.30, 3.62)**
Persisted on PrEP 1 month	80 (of 193; 41%)	34 (of 83; 41%)	1.00 (0.59, 1.68)	0.90 (0.53, 1.54)
Persisted on PrEP through 4 months	31 (of 193; 16%)	15 (of 83; 18%)	1.16 (0.59, 2.30)	1.08 (0.55, 2.16)

*Note*: No return for study visit is equivalent to discontinued PrEP for this study definition. Bold *p*≤0.05.

Abbreviations: CI, confidence interval; PrEP, pre‐exposure prophylaxis; STI, sexually transmitted infection.

^a^ Adjusted for participant age and sex.

^b^ Persisted at both 1‐ and 4‐month visits; confirmed with repeat prescription, through to 4 months.

### 3.7 HIV seroconversion

We identified three participants who seroconverted after initiating PrEP at baseline. Two participants were diagnosed at 1 month, and one participant was diagnosed at the 4‐month follow‐up visit. Of those who seroconverted, one self‐reported to be using PrEP with some missed doses in the past 30 days, and two reported they had discontinued PrEP use for over 1 month. No other social or health harms were identified in the study.

## 4 DISCUSSION

In our randomized study, STI POC testing alone did not affect initial PrEP initiation, though participants who received a POC STI test had moderately improved PrEP persistence through 4 months (. In secondary analysis, participants who received syndromic STI treatment or STI treatment in those diagnosed with a POC STI test had an increased likelihood of PrEP initiation compared with those who were not diagnosed or treated. Our study demonstrated it was feasible and acceptable to integrate STI testing and syndromic management into ongoing SRH and PrEP services in a mobile, community setting with high proportions of STI treatment. We demonstrated that STI diagnosis and subsequent treatment can serve as pivotal moments to encourage individuals to engage in preventive HIV measures such as PrEP [1] and support the recommendation for integrated HIV/STI and SRH services for young people in high HIV‐incidence communities.

Participants who received POC STI testing, and those diagnosed and treated with an STI in either study arm, were moderately more likely to persist on PrEP through 4 months (25% vs. 19% and 19% vs. 14%, respectively). These findings provide valuable insights into the complex interplay between STI management and PrEP uptake and persistence among youth seeking SRH care in a high STI and HIV prevalence community. The results reveal a high prevalence of STIs among the youth population, with a notable gender disparity, echoing findings from prior studies that highlight the high burden of STIs among young females [4, 20, 21].

POC STI testing was effective in diagnosing and treating STIs on the same day in a high STI‐prevalence community. In this community‐based, very accessible PrEP programme, there is a high initiation rate of PrEP at baseline which reflects the initial acceptability of PrEP among participants. The initiation rate was boosted after STI treatment leading to three‐quarters of young people coming to the SRH service starting oral PrEP. Diagnosis or treatment with an STI may underpin the perception of one's risk of HIV acquisition. However, PrEP persistence at 4 months was low for all groups, with fewer than one‐fifth continuing PrEP even within this community‐based mobile delivery model. Treatment for an STI or STI syndrome was associated with an increase in PrEP initiation and moderate association with 4‐month PrEP persistence. Integration of STI management may improve PrEP initiation and persistence among young people. This finding is consistent with previous research, which highlights the ongoing challenges in ensuring PrEP persistence over time [6, 11].

This finding aligns with previous studies that suggest immediate and effective STI treatment reinforces the importance of PrEP, likely due to increased engagement with healthcare services and heightened awareness of personal health risks [1, 7]. A recent study by De Voux et al. in Cape Town demonstrated improved PrEP persistence among pregnant women treated with an STI [22]. Previous studies have, however, demonstrated mixed results of the effect of integrating STI testing into PrEP care [11, 21]. Moreover, our study contributes to the growing evidence that integration of STI and HIV services can leverage resources and enhance the effectiveness of both interventions [15, 22]. This underscores the synergistic potential of integrating STI screening and treatment into PrEP programmes, not only to reduce STI burden but also to optimize PrEP adherence. Future research should explore scalable models for routine STI testing within PrEP services, assess long‐term adherence outcomes and investigate behavioural mechanisms driving continued PrEP use following STI diagnosis and treatment.

A notable limitation of our study is that we recruited young people who visited a well‐established adolescent SRH service location known to be adolescent‐focused and welcoming. Thus, our sample may have limited generalizability when compared to other PrEP clinic groups. The study's follow‐up period was short, limiting our ability to assess long‐term PrEP persistence; however, persistence challenges occur early in PrEP programmes of young people, and this appears to be the area to impact. The persistence outcomes may be influenced by self‐report bias; thus, we used prescription to ascertain this data. Study retention may have differed by study intervention which may over‐report the effect of STI testing on PrEP use. Despite the clear procedural differences between POC STI testing and syndromic management, the lack of significant differences in PrEP initiation and persistence on PrEP suggests that other factors beyond the testing method, such as overall engagement with healthcare services, may have been equally important across both study arms [22, 23].

Future research should explore the long‐term impact of POC STI testing on PrEP persistence in populations who are at risk of HIV acquisition, considering additional factors such as adherence support, behavioural interventions and integration with other preventive services and improved STI treatment services. Studies should also aim to understand the barriers to PrEP persistence beyond the initial months, focusing on strategies to sustain long‐term use. Tailored interventions that also consider client needs and behaviours may enhance both STI management and PrEP outcomes [24].

## 5 CONCLUSIONS

Our study demonstrates that same‐day aetiologic STI testing, by itself, did not improve PrEP initiation nor persistence among South African youth. However, same‐day STI treatment, whether following STI testing or syndromic management, did improve both the proportion who initiated PrEP and persisted at 4 months. These findings provide valuable insights for policymakers and healthcare providers aiming to optimize SRH services for high‐risk populations. Future research should investigate strategies to integrate STI and PrEP services to enhance their combined effectiveness.

## COMPETING INTERESTS

The authors have no competing interests to declare that are relevant to the content of this article.

## AUTHORS’ CONTRIBUTIONS

Conceptualization: L‐GB, DJD, ER and PM. Project administration: LF, PM, BL, KL and AK. Data curation: BL, KL and LF. Analysis: KL and DJD. Writing—original draft preparation: DJD and LF. Writing—review and editing: L‐GB and DJD. Funding acquisition: L‐GB, DJD and ER. Supervision: L‐GB, DJD and ER.

## FUNDING

Funding was provided by The Gates Foundation to Desmond Tutu HIV Foundation. The funders had no role in the study design, data collection and analysis, nor will they have any role in manuscript preparations or publication decisions.

## Data Availability

Study protocol and de‐identified data presented in this manuscript will be shared upon reasonable request and receipt of a completed data request form from the corresponding author.
