# Correction to Evaluation of point‐of‐care diagnostics for sexually transmitted infection on oral PrEP initiation and persistence among young people in South Africa: a randomized controlled study

**DOI:** 10.1002/jia2.26519

**Published:** 2025-06-05

Joseph Davey, D., Fynn, L., Rousseau, E., Macdonald, P., Leonard, B., Lebelo, K., Kolisa, A., Little, F. and Bekker, L.‐G. (2025), Evaluation of point‐of‐care diagnostics for sexually transmitted infection on oral PrEP initiation and persistence among young people in South Africa: a randomized controlled study. J Int AIDS Soc., 28: e26488. https://doi.org/10.1002/jia2.26488

In the article, one of the author names was incorrect and should be changed to *Keitumetse Lebelo* from Keitumese Lebelo.

The online version of the article was corrected.

We apologize for this error.

